# Efficient partition of integer optimization problems with one-hot encoding

**DOI:** 10.1038/s41598-019-49539-6

**Published:** 2019-09-10

**Authors:** Shuntaro Okada, Masayuki Ohzeki, Shinichiro Taguchi

**Affiliations:** 1 Electronics R & I Division, DENSO CORPORATION, Tokyo, 108-0075 Japan; 2 Graduate School of Information Sciences, Tohoku University, Sendai, 980-8579 Japan; 3 Institute of Innovative Research, Tokyo Institute of Technology, Yokohama, 226-8503 Japan; 4 Sigma-i Co. Ltd., Tokyo, 108-0075 Japan

**Keywords:** Computer science, Information theory and computation

## Abstract

Quantum annealing is a heuristic algorithm for solving combinatorial optimization problems, and hardware for implementing this algorithm has been developed by D-Wave Systems Inc. The current version of the D-Wave quantum annealer can solve unconstrained binary optimization problems with a limited number of binary variables. However, the cost functions of several practical problems are defined by a large number of integer variables. To solve these problems using the quantum annealer, integer variables are generally binarized with one-hot encoding, and the binarized problem is partitioned into small subproblems. However, the entire search space of the binarized problem is considerably larger than that of the original integer problem and is dominated by infeasible solutions. Therefore, to efficiently solve large optimization problems with one-hot encoding, partitioning methods that extract subproblems with as many feasible solutions as possible are required. In this study, we propose two partitioning methods and demonstrate that they result in improved solutions.

## Introduction

The combinatorial optimization problems aim to minimize cost functions defined by discrete variables, and these problems often have significant real-world applications. In general, the cost function of a combinatorial optimization problem can be expressed as the Hamiltonian of a classical Ising model^1^. Therefore, many algorithms for solving combinatorial optimization problems have been inspired by physics. Simulated annealing (SA)^2^ is one of the most famous algorithms, employing thermal fluctuations to escape local minima. In contrast to SA, quantum annealing (QA)^3^ is a method that exploits quantum fluctuations and the resulting tunneling effect. A popular research topic involves evaluating whether it is more advantageous to employ quantum effects than thermal fluctuations, and numerous studies have been conducted on this topic^4–9^. In addition, the recent development of a commercial quantum annealer by D-Wave Systems Inc^10^. has attracted many companies and researchers. The performance of QA has been experimentally studied using the quantum annealer and compared with that of SA^11–13^, and several companies have demonstrated the applicability of the annealer to practical problems^14–30^.

The time-dependent Hamiltonian of QA is given as follows

1 $$\hat{H}(t)=A(t){\hat{H}}_{{\rm{q}}}+B(t){\hat{H}}_{0},$$

where ${\hat{H}}_{0}$ is the target Hamiltonian representing the cost function, and ${\hat{H}}_{{\rm{q}}}$ denotes the quantum fluctuation term for which the ground state is trivial. The initial values of the coefficients are set to *A*(0) = 1 and *B*(0) = 0, and the system is prepared in the trivial ground state determined by ${\hat{H}}_{{\rm{q}}}$. Then, the strength of the quantum fluctuation is reduced toward zero, and the coefficients are set to *A*(*τ*) = 0 and *B*(*τ*) = 1 at the end of QA, where *τ* is the annealing time. The dynamics of the system is described by the Schrödinger equation:

2 $$i\frac{d}{dt}\psi (t)=\hat{H}(t)\psi (t),$$

where *ψ*(*t*) is the state vector of the system, and *ℏ* is set to 1 for simplicity. Given that the coefficients change sufficiently slowly, the adiabatic theorem^31^ ensures that the system remains close to the instantaneous ground state of the time-dependent Hamiltonian. Thus, by setting the annealing time *τ* to be sufficiently large, the ground states of the target Hamiltonian ${\hat{H}}_{0}$ can be obtained with high probability.

The current version of the D-Wave quantum annealer (D-Wave 2000Q) implements transverse-magnetic-field QA, for which the quantum fluctuation is given as follows:

3 $${\hat{H}}_{{\rm{q}}}=-\,\mathop{\sum }\limits_{i=1}^{{N}_{{\rm{q}}}}{\hat{\sigma }}_{i}^{(x)},$$

where *N*_q_ denotes the number of qubits. The quantum annealer can handle a cost function as follows:

4 $${\hat{H}}_{0}=\sum _{(i,j)\in {\rm{chimera}}}{J}_{ij}{\hat{\sigma }}_{i}^{(z)}{\hat{\sigma }}_{j}^{(z)}+\mathop{\sum }\limits_{i=1}^{{N}_{{\rm{q}}}}{h}_{i}{\hat{\sigma }}_{i}^{(z)},$$

where the interactions between qubits are restricted to the Chimera graph^32^, which is a 16 × 16 grid of complete bipartite graphs *K*_4,4_ in D-Wave 2000Q. It should be noted that the number of operable qubits is less than *N*_q_ = 2,048 due to defects in the qubits and connectivities.

Due to the limited number of available qubits, large optimization problems cannot be solved directly using the D-Wave quantum annealer. In real settings, large problems are partitioned into subproblems that can be handled by the quantum annealer. The subproblems are iteratively optimized by the quantum annealer, and the optimization result is used to improve the current solution^33–35^. A cluster of spins in the subproblem is simultaneously updated in this scheme; this iterative method is a type of large-neighborhood local search algorithm^36^. Although these algorithms can be performed using classical computers, subproblems are fundamentally restricted to tree structures that are solvable in polynomial time by belief propagation or dynamic programming^37–40^. Therefore, using the quantum annealer is advantageous if it can solve subproblems with many closed loops more efficiently than classical algorithms. Furthermore, solving subproblems that are as large as possible is essential for improving solution accuracy^41^. The size of subproblems that can be embedded into the quantum annealer strongly depends on the quality of the minor embedding, in particular for problems with few interactions. Because subproblems must be iteratively embedded, fast algorithms for embedding larger subproblems are required for exploiting the potential of the quantum annealer. Although complete-graph embedding^42–44^ can be used for problems with dense interactions, a subproblem-embedding algorithm, that was developed in a previous study^41^, may be effective in improving the solution accuracy of sparse problems.

**Figure 1:**
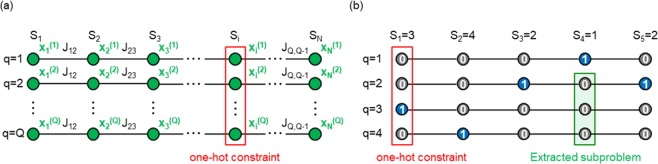
(**a**) Problem graph of Eq. (7). Vertices and edges represent binary variables ${x}_{i}^{(q)}$ and the interactions between them, respectively. Although the penalty term generates fully connected vertical interactions between ${x}_{i}^{(q)}$ and ${x}_{i}^{(q^{\prime} )}$, these are not shown for simplicity. *Q* binary variables ${\{{x}_{i}^{(q)}\}}_{q=1,2,...,Q}$ are assigned to each *S*_*i*_, and the total number of binary variables is *NQ*. Although there exist 2^*NQ*^ configurations of the binary variables, only *Q*^*N*^ configurations satisfy the one-hot constraint. (**b**) Example of an undesirable partition, where binary variables enclosed by the green rectangle are extracted as a subproblem. The binary variables colored blue represent the tentatively selected components in the current solution. Superior feasible solutions cannot be explored by optimizing the extracted subproblem.

In addition, the quantum annealer requires the cost function to be represented in the form of a quadratic unconstrained binary optimization (QUBO) problem or Ising model; however, many cost functions in practical problems are defined by integer variables. The binarization of integer variables is generally achieved using one-hot encoding^1^. For example, suppose that we wish to solve the following integer optimization problem with *N* integer variables {*S*_*i*_}_*i* = 1,2,...,*N*_:

5 $$\mathop{{\rm{\arg }}\,{\rm{\min }}}\limits_{\{{S}_{i}\}}\mathop{\sum }\limits_{i=1}^{N-1}{J}_{i,i+1}\delta ({S}_{i},{S}_{i+1}),$$

where *S*_*i*_ ∈ (1, 2, ..., *Q*), *Q* is the number of components, *J*_*i*,*i*+1_ is an interaction between *S*_*i*_ and *S*_*i*+1_, and *δ* is the Kronecker delta function. The integer variables {*S*_*i*_}_*i* = 1,2,...,*N*_ can be binarized by one-hot encoding as follows:

6 $$\mathop{{\rm{\arg }}\,{\rm{\min }}}\limits_{\{{x}_{i}^{(q)}\}}\mathop{\sum }\limits_{i=1}^{N-1}{J}_{i,i+1}\mathop{\sum }\limits_{q=1}^{Q}{x}_{i}^{(q)}{x}_{i+1}^{(q)}\,{\rm{s}}.\,{\rm{t}}.\,\mathop{\sum }\limits_{q=1}^{Q}{x}_{i}^{(q)}=1,$$

where ${x}_{i}^{(q)}\in (0,1)$ is a binary variable that is assigned to component *q* of *S*_*i*_, and ${x}_{i}^{(q)}=1$ indicates that component *q* is selected for *S*_*i*_. In addition, feasible solutions are constrained to configurations in which exactly one component is selected for each *S*_*i*_. Subsequently, a penalty term is introduced to obtain the following unconstrained form:

7 $${H}_{0}=\mathop{\sum }\limits_{i=1}^{N-1}{J}_{i,i+1}\mathop{\sum }\limits_{q=1}^{Q}{x}_{i}^{(q)}{x}_{i+1}^{(q)}+\lambda \mathop{\sum }\limits_{i=1}^{N}{(\mathop{\sum }\limits_{q=1}^{Q}{x}_{i}^{(q)}-1)}^{2},$$

where the second term formulates the penalty term which is introduced to extract feasible solutions satisfying the constraint ${\sum }_{q=1}^{Q}{x}_{i}^{(q)}=1$, which we call the *one-hot constraint*, and parameter *λ* controls the strength of the penalty term. By setting parameter *λ* to a sufficiently large value, the ground states of the original integer optimization problem (Eq. (5)) are correctly encoded. However, the performance of the D-Wave quantum annealer is significantly affected by noise and intrinsic control errors when *λ* is larger than necessary. Therefore, to obtain highly accurate solutions, we must explore an appropriate value of *λ*, which is a tedious task for optimization under the one-hot constraint. In addition, the entire search space of the binarized optimization problem (Eq. (7)) is dominated by infeasible solutions. Figure 1(a) presents the problem graph of Eq. (7). In this figure, vertices and edges represent binary variables and the interactions between them, respectively. *Q* binary variables ${\{{x}_{i}^{(q)}\}}_{q=1,2,...,Q}$ are assigned to each *S*_*i*_, and the total number of binary variables is *NQ*. Although the number of configurations of the binary variables is 2^*NQ*^, the number of feasible solutions is only *Q*^*N*^. Therefore, to efficiently solve large optimization problems under the one-hot constraint using the quantum annealer, partitioning methods are required for extracting subproblems with as many feasible solutions as possible. A simple example of an undesirable partition is depicted in Fig. 1(b). Here, suppose that we wish to improve the current solution presented in Fig. 1(b) and that the three binary variables enclosed by the green rectangle are extracted as the subproblem. In this case, superior feasible solutions cannot be explored by optimizing the subproblem because only the current solution in the subproblem satisfies the one-hot constraint. To the best of our knowledge, the partitioning method proposed by Nishimura *et al*.^30^ is the first to focus on the one-hot constraint. This method is applicable to the double-constrained problems ${\sum }_{q}{x}_{i}^{(q)}=1$ and ${\sum }_{i}{x}_{i}^{(q)}=1$, such as the assignment problem and the traveling salesman problem. However, the extracted subproblems still contain infeasible solutions for which parameter *λ* must be adjusted. In this study, we propose two partitioning methods applicable to problems whose cost function involves a single one-hot constraint, as illustrated in Eq. (7). The first method is similar to the partition proposed by Nishimura *et al*., while the second method extracts subproblems comprising only feasible solutions and does not require adjusting parameter *λ*. The performance of the proposed methods is assessed for several Potts models, which are generalized Ising models whose cost function is defined by integer variables^45^. We demonstrate that the proposed methods efficiently obtain superior solutions.

## Results

In this section, we propose efficient partitioning methods for solving large integer optimization problems under the one-hot constraint. In addition, we assess the performance of the proposed methods for several Potts models.

### Proposed methods

**Figure 2:**
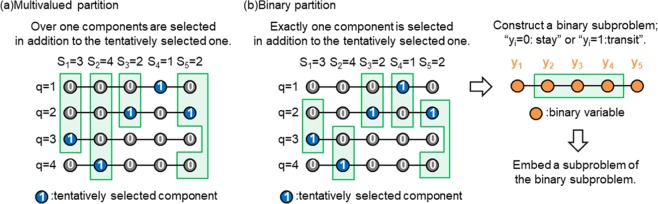
Proposed methods for finding superior solutions to large optimization problems under the one-hot constraint.

We propose two partitioning methods: a multivalued partition and a binary partition. These methods are summarized in Fig. 2. Both methods extract a subproblem that involves binary variables assigned to the tentatively selected components for each *S*_*i*_. The resulting subproblems include feasible solutions other than the current solution.

The multivalued partition extracts a subproblem with two or more components for each *S*_*i*_, as illustrated in Fig. 2(a). In addition to the tentatively selected component, the multivalued partition randomly selects one or more components for each *S*_*i*_, and then extracts a subproblem that comprises the binary variables assigned to the selected components. The extracted subproblem involves feasible solutions other than the current solution, and the randomly selected components are explored for each *S*_*i*_ by optimizing the subproblem. However, the extracted subproblem still contains infeasible solutions, and the penalty term remains in the cost function of the subproblem. This partitioning method is similar to the partition proposed by Nishimura *et al*. Although subproblems are embedded using complete-graph embedding in the study by Nishimura *et al*.^30^, we employed the subproblem-embedding algorithm developed in our previous paper^41^. Details of achieving a multivalued partition using the subproblem-embedding algorithm are provided in the Methods section.

The binary partition is summarized in Fig. 2(b). In addition to a tentatively selected component, the binary partition randomly selects exactly one component for each *S*_*i*_. Subsequently, new binary variables {*y*_*i*_}_*i*__ = 1,2,...,*N*_ that represent “stay in the tentatively selected component (*y*_*i*_ = 0)” or “transit to the randomly selected component (*y*_*i*_ = 1)” are introduced for each *S*_*i*_, and a binary subproblem is constructed whose cost function is defined by {*y*_*i*_}_*i* = 1,2,...,*N*_. The cost function of the binary subproblem is derived in the Methods section. Thereafter, a subproblem of the binary subproblem is embedded into the D-Wave quantum annealer by the subproblem-embedding algorithm^41^. Here, the cost function of the binary subproblem does not involve the penalty term because all solutions in the binary subproblem are feasible. Therefore, the binary partition does not require adjusting parameter *λ*. In addition, a larger number of binary variables can be embedded into the D-Wave quantum annealer because the penalty term, which generates fully connected interactions between ${x}_{i}^{(q)}$ and ${x}_{i}^{(q\text{'})}$, is not involved. Consequently, the number of feasible solutions involved in the embedded subproblem is significantly increased using the binary partition. The binary subproblem can be regarded as one of the simplest cases of optimization under the half-hot constraint^46^. The penalty term of the half-hot constraint is given by

8 $$\lambda \mathop{\sum }\limits_{i=1}^{N}{(\mathop{\sum }\limits_{q=1}^{Q}{x}_{i}^{(q)}-\frac{Q}{2})}^{2},$$

and *Q*/2 components are extracted. The half-hot constraint is proposed to avoid the difficulty caused by the longitudinal magnetic field in the penalty term of the one-hot constraint. This difficulty is avoidable using the binary partition, which may contribute to improving solution accuracy. A disadvantage of the binary partition is that only two components are considered for each integer variable. As demonstrated in the following subsection, this leads to poor performance for the ferromagnetic Potts model.

### Performance assessment

The performance of the proposed methods is evaluated for the following four types of Potts models on a cubic lattice with 10 × 10 × 10 integer variables: the ferromagnetic, anti-ferromagnetic, Potts glass^47^ and Potts gauge glass^48,49^ models. While the ground states of the ferromagnetic and anti-ferromagnetic Potts models are trivial, it is generally difficult to obtain the ground states of the Potts glass and Potts gauge glass models due to competing interactions.

The cost function is given by

9 $${H}_{0}=\sum _{ < i,j > }{J}_{ij}\delta ({S}_{i},{S}_{j}+{\Delta }_{ij}),$$

where *Q* is set to 4, *S*_*i*_ ∈ (1, 2, 3, 4), Δ_*ij*_ ∈ (0, ±1), *δ* is the Kronecker delta function, and *J*_*ij*_ represents the interaction between the nearest neighbors on the cubic lattice with the periodic boundary condition. The cost function is represented in QUBO form using the one-hot constraint as follows:

10 $${H}_{0}=\sum _{ < i,j > }{J}_{ij}\mathop{\sum }\limits_{q=1}^{4}{x}_{i}^{(q)}{x}_{j}^{(q-{\Delta }_{ij})}+\lambda \mathop{\sum }\limits_{i=1}^{1000}{(\mathop{\sum }\limits_{q=1}^{4}{x}_{i}^{(q)}-1)}^{2}.$$

**Figure 3:**
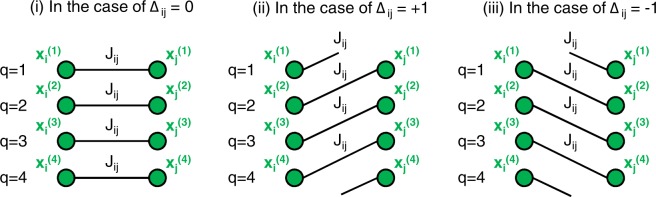
Local interactions generated by the first term of Eq. (10). Δ_*ij*_ ≠ 0 causes interactions between different components.

Parameters *J*_*ij*_, Δ_*ij*_, and *λ* in each model are presented in Table 1. Δ_*ij*_ ≠ 0 generates interactions between different components in the Potts gauge glass model, and Fig. 3 illustrates the local interactions generated by the first term of Eq. (10). Although it is generally difficult to determine an appropriate value of *λ* a priori, we can derive the lower bound of *λ* to correctly encode the original optimal solutions for the ferromagnetic and anti-ferromagnetic Potts models. The lower bound strongly depends on whether there exist infeasible solutions that set the first term of Eq. (10) to a smaller value than that of the original optimal solutions. *λ* > 0 is sufficient if such infeasible solutions do not exist; however, a sufficiently large value of *λ* is necessary if such infeasible solutions exist. For the ferromagnetic Potts model, the first term of Eq. (10) for an infeasible solution with ${x}_{i}^{(1)}={x}_{i}^{(2)}=1,{x}_{i}^{(q\ge 3)}=0$ is lower than that of the original optimal solution (e.g., ${x}_{i}^{(1)}=1,{x}_{i}^{(q\ge 2)}=0$) by 3*N*. Because the second term in Eq. (10) increases by *Nλ* in this infeasible solution, *λ* > 3 is required. In this study, we set *λ* = 3.3 because an unnecessarily large value is not preferable, as mentioned in the Introduction section. While for the anti-ferromagnetic Potts model, the original optimal solutions minimize the first term of Eq. (10). Therefore, *λ* > 0 is sufficient, and we set *λ* = 1.0, which is the same value as *J*_*ij*_ in the first term. However, for the Potts glass and Potts gauge glass models, the lower bound cannot be derived because the original optimal solutions are not trivial. At least, by setting *λ* > 3, we can restrict energy changes caused by a single-spin flip from the optimal solutions to be a positive value, and *λ* is set to 3.3 in this study.

**Table 1:** Parameter settings of cost function Eq. (10).

Model	*J*_*ij*_	Δ_*ij*_	*λ*
Ferromagnetic Potts model	−1	0	3.3
Anti-ferromagnetic Potts model	+1	0	1.0
Potts glass model	+1 (50%) or −1 (50%)	0	3.3
Potts gauge glass model	−1	0 (50%) or +1 (25%) or −1 (25%)	3.3

**Figure 4:**
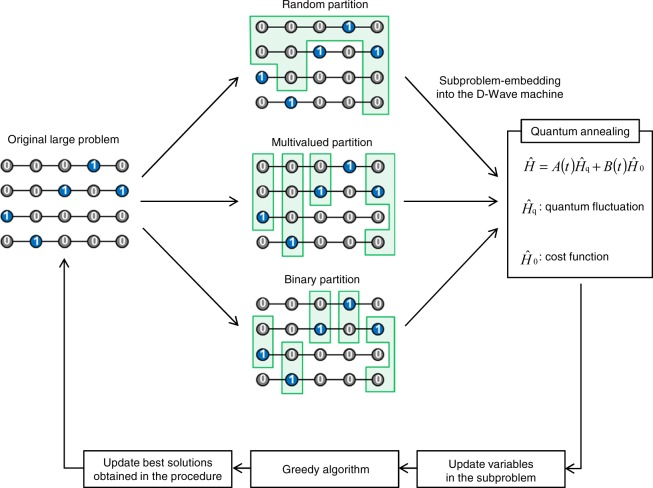
Optimization process demonstrated in this study. The solution accuracy is evaluated for the three partitioning methods.

The optimization process demonstrated in this study is illustrated in Fig. 4. The original large problem is partitioned using three partitioning methods: random, multivalued, and binary partitions. The random partition does not address whether an extracted subproblem contains feasible solutions for each *S*_*i*_. A subproblem-embedding algorithm proposed in the literature^41^ is used for embedding a subproblem into the D-Wave quantum annealer with defects in qubits and the interactions between them (details on embeddings are provided in the Methods section). After optimizing the embedded subproblem by the D-Wave quantum annealer under the parameter settings provided in Table 2, the variables in the subproblem are replaced by the best solution among the 1,000 solutions obtained using the quantum annealer. Subsequently, a greedy algorithm is executed by a conventional digital computer to recover the one-hot constraint and obtain an exact (local) minimum. In this algorithm, if there exist integer variables violating the one-hot constraint, the constraint is first recovered by extracting the integer variables and selecting exactly one component that minimizes the local energy for each integer variable. Then, an integer variable is randomly selected, and the tentatively selected component is replaced with one that minimizes the local energy. Refining the current solution is completed when all local energies are minimized. Finally, the best solution obtained in the procedure is updated, and the above processes are iterated. We then compare the solution accuracy for the three partitioning methods.

**Table 2:** Parameter settings of D-Wave quantum annealer.

Parameter	Value
solver	D-Wave 2000Q_2
annealing time	20[*μ*s]
Num_reads	1,000
auto_scale	True
postprocess	none

**Figure 5:**
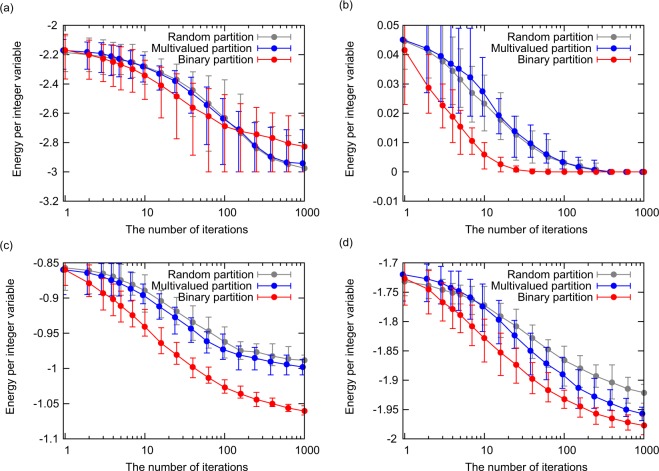
Energies obtained using the three partitioning methods. The average, maximum, and minimum energies for 16 trials are plotted. (**a**) Ferromagnetic Potts model. (**b**) Anti-ferromagnetic Potts model. (**c**) Potts glass model. (**d**) Potts gauge glass model.

Figure 5 represents the energies obtained by the three partitioning methods. The average, maximum, and minimum energies for 16 trials are plotted, and the same 16 initial states are used for each partitioning method. The horizontal axis represents the number of iterations, which is the number of subproblem optimizations performed by the D-Wave quantum annealer. The plot for the multivalued partition is shifted slightly to the left to avoid overlap with other plots. Figure 5(a,b) illustrate the energies obtained for the ferromagnetic and anti-ferromagnetic Potts models, respectively. The ground states of these models are trivial, and the minimum energy is −3 and 0 for the ferromagnetic and anti-ferromagnetic Potts models, respectively. Although the multivalued partition is expected to solve large optimization problems more efficiently than the random partition, the performances of the random and multivalued partitions are almost identical. The performance of the binary partition differs from the other methods, however; it is the lowest for the ferromagnetic Potts model, and the highest for the anti-ferromagnetic Potts model. Figure 5(c,d) present the energies obtained for the Potts glass and Potts gauge glass models, respectively. As expected, superior solutions are obtained with a smaller number of iterations using the multivalued partition rather than the random partition, in particular for the Potts gauge glass model. Of the three partitioning methods, the binary partition shows the highest performance for both the Potts glass and Potts gauge glass models.

## Discussion

In this section, we discuss the differences between the three partitioning methods. The following three questions arise from the results presented in the previous section.

- Why is the multivalued partition not superior to the random partition for the ferromagnetic and anti-ferromagnetic Potts models?
- Why is the performance of the binary partition the lowest for the ferromagnetic Potts model?
- Why does the binary partition exhibit the highest performance for all models except for the ferromagnetic Potts model?

**Figure 6:**
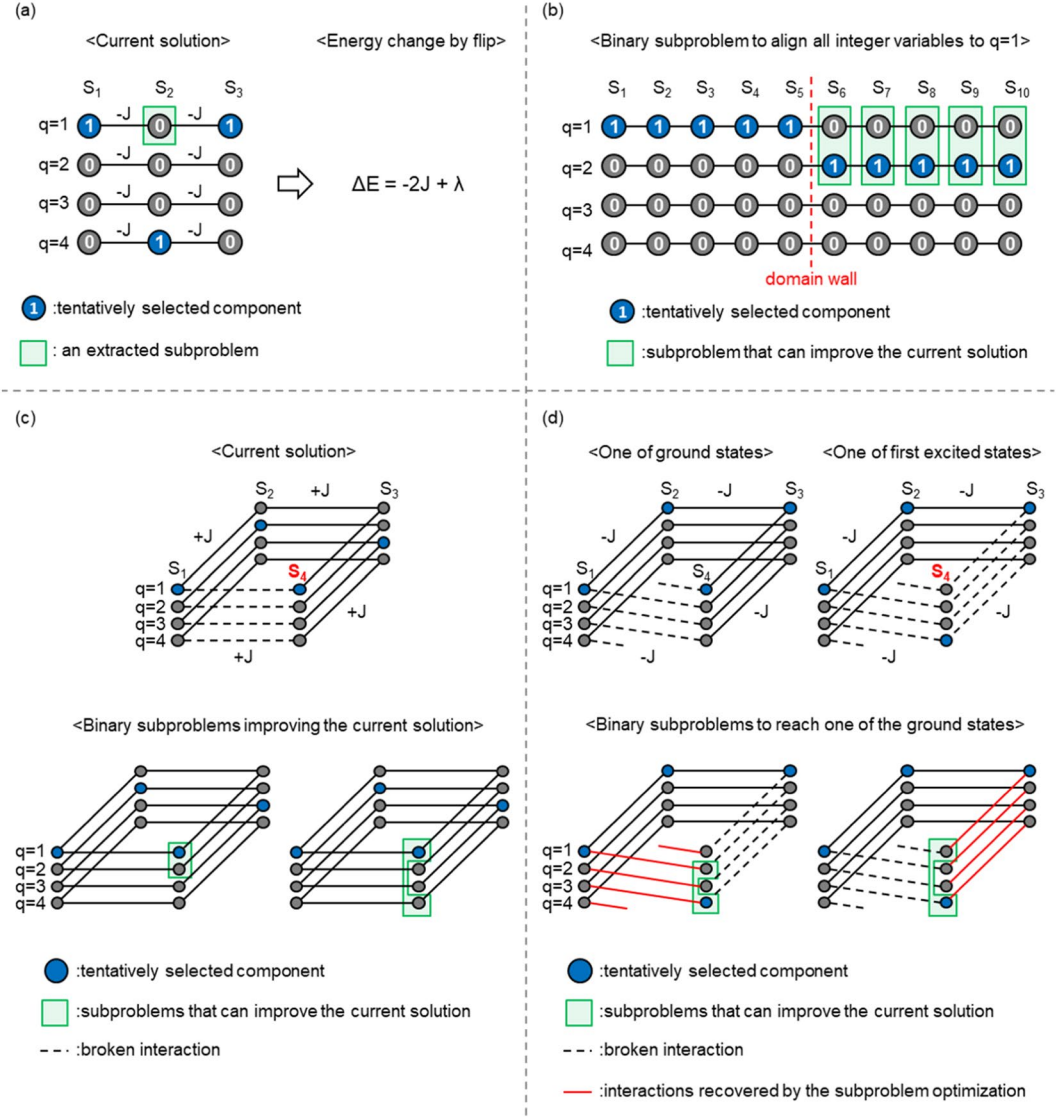
Discussion on results. Vertices and edges represent binary variables $\{{x}_{i}^{(q)}\}$ and the interactions between them, respectively. Binary variables colored blue are tentatively selected components, and binary variables enclosed by green rectangles are the extracted subproblem. (**a**) Simple example of the random partition that can reduce the energy despite violating the one-hot constraint. (**b**) One of the first excited states commonly observed in the optimization of the ferromagnetic Potts model. To align all integer variables {*S*_*i*_}_*i* = 1,2,...,10_ to *q* = 1, component *q* = 1 must be selected for variables *S*_6_, ..., *S*_10_. (**c**) Local interactions in the anti-ferromagnetic Potts model. The two binary subproblems can improve the current solution. (**d**) Simple example of the Potts gauge glass model. One interaction is broken in the ground state due to competing interactions between different components. Two binary subproblems can improve the first excited state. Broken interactions are represented by dashed lines, while interactions recovered by optimizing the binary subproblem are represented by red lines.

A possible answer to the first question is that, for the ferromagnetic and anti-ferromagnetic Potts models, improved feasible solutions can be obtained through infeasible solutions because there likely exist infeasible solutions whose energy is lower than that of a current feasible solution in a neighbor. Figure 6(a) presents a simple example for the one-dimensional ferromagnetic Potts model. We assume that the binary variable enclosed by the green rectangle is extracted as a one-variable subproblem, which is one of the simplest cases of the random partition. The energy change caused by flipping the extracted binary variable is −2*J* + *λ* because two interactions are simultaneously recovered (−2*J*) and the one-hot constraint is violated (+*λ*). If *λ* < 2*J*, flipping the binary variable decreases the energy despite violating the constraint. It should be noted that *λ* > *J* is sufficient to correctly encode the ground states of the one-dimensional ferromagnetic Potts model. This is because the energy of the lowest-energy infeasible states, in which two components are commonly selected for each *S*_*i*_, is −2*NJ* + *Nλ* and must be larger than that of the ground states (−*NJ*). Consequently, if *λ* is appropriately tuned (*J* < *λ* < 2*J*), the current solution is updated to the infeasible solution by optimizing the subproblem, and superior feasible solutions are obtained via the infeasible solution. Whether energy changes become negative or not in spite of violating the one-hot constraint strongly depends on the number of simultaneously recovered interactions. For the ferromagnetic and anti-ferromagnetic Potts models without competing interactions, many interactions can be simultaneously recovered by violating the one-hot constraint. As a result, the multivalued partition is not effective in improving the solution accuracy for these models. In contrast, for the Potts glass and Potts gauge glass models with competing interactions, the performance of the multivalued partition is superior to that of the random partition.

In answer to the second question, subproblems that can eliminate domain walls are rarely extracted by the binary partition. Figure 6(b) presents one of first excited states, which is commonly observed in the optimization of the ferromagnetic Potts model. The 10 variables in Fig. 6(b) are divided into two domains: five variables *S*_1_, ..., *S*_5_ are aligned to *q* = 1 in one domain, while the remaining five variables *S*_6_, ..., *S*_10_ are aligned to *q* = 2 in the other domain. The boundary between the domains is referred to as the domain wall. To improve the current solution, an extracted subproblem must contain one of the ground states because the current solution is the first excited state. For example, to align all integer variables {*S*_*i*_}_*i* = 1,2,...,10_ to *q* = 1, component *q* = 1 must be selected for integer variables *S*_6_, ..., *S*_10_. The probability of component *q* = 1 being selected for *S*_6_, ..., *S*_10_ is (1/3)^5^ = 1/243 because, in addition to the tentatively selected component, the binary partition randomly selects one component for each *S*_*i*_. This probability exponentially decreases with respect to the number of variables, and the extraction of only two components is not suitable for the ferromagnetic Potts model. We further conjecture that the binary partition exhibits poor performance for optimization problems containing ferromagnetically ordered domains, and that the concomitant use of the binary and multivalued partitions may be preferable for such problems.

The answer to the third question is that there exist several binary subproblems that can improve the current solution. Figure 6(c) illustrates the local interactions in the anti-ferromagnetic Potts model. The current solution is one of the first excited states in which the local energy with respect to *S*_1_ and *S*_4_ is not minimized, which we say “the interaction between *S*_1_ and *S*_4_ is *broken*” in this paper. Assuming that the integer variable *S*_4_ is updated to improve the current solution, there are two binary subproblems that can improve the current solution. Therefore, the disadvantage of the binary partition, which is that only two components are considered for each integer variable, is mitigated for the optimization of the anti-ferromagnetic Potts model. We can thus exploit the advantages of the binary partition, which are that the extracted subproblems contain a larger number of feasible solutions and that the adjustment of parameter *λ* is not required. This is also the case for the Potts glass and Potts gauge glass models, in which competing interactions generate several binary subproblems that improve the current solution. Figure 6(d) presents a simple example for the Potts gauge glass model. One of the ground states and first excited states are illustrated at the top of Fig. 6(d), where the interaction depicted by a dashed line represents the broken interaction. There exist no configurations that minimize all of the local energies due to the competing interaction between different components, and one interaction is broken even in the ground state. Suppose that we update integer variable *S*_4_ to improve the current solution in the first excited state, then there are two binary subproblems that can improve the current solution, as illustrated at the bottom of Fig. 6(d). One subproblem recovers the interaction between *S*_1_ and *S*_4_, while the other subproblem recovers the interaction between *S*_3_ and *S*_4_. The competing interactions generate two binary subproblems that improve the current solution, and each subproblem recovers different interactions. Thus, the disadvantage of the binary partition is mitigated as long as *Q* is not very large. It should be noted that although the number of binary subproblems that improve the current solution increases as *Q* is increased for the anti-ferromagnetic Potts model, this number does not increase for the Potts gauge glass model.

## Conclusion

In this study, we proposed two partitioning methods to efficiently solve large optimization problems under the one-hot constraint using the D-Wave quantum annealer. The performance of the proposed methods was assessed for the ferromagnetic, anti-ferromagnetic, Potts glass, and Potts gauge glass models. Of the three partitioning methods, the binary partition showed the highest performance for all models except for the ferromagnetic Potts model. The advantages of the binary partition are that it enables embedding a larger number of binary variables and does not require adjusting parameter *λ*. However, its disadvantage is that only two components are considered for each integer variable. Although this disadvantage leads to poor performance for the ferromagnetic Potts model, the effect is mitigated for optimization problems that have many binary subproblems improving the current solution, such as the anti-ferromagnetic Potts model, and for optimization problems with competing interactions. Although the multivalued partition exhibits a better performance than the random partition for Potts glass and Potts gauge glass models, we did not identify problems for which the multivalued partition is most suitable. Future studies should focus on constructing algorithms that can efficiently solve the ferromagnetic Potts model using the binary partition. In addition, the performance of the proposed methods should be assessed for various optimization problems, such as the graph coloring problem whose cost function is represented as the Hamiltonian of the anti-ferromagnetic Potts model.

## Methods

Details on partitioning and embedding are provided in this section.

### Subproblem-embedding algorithm

In this subsection, we briefly explain the subproblem-embedding algorithm developed in a previous study^41^. This algorithm aims to quickly find minor embeddings of subproblems to efficiently implement large-neighborhood local searches using the D-Wave quantum annealer.

Given a large optimization problem whose cost function is represented in QUBO form, this algorithm embeds binary variables one by one into the Chimera graph. After a randomly selected binary variable is embedded into the Chimera graph first, this algorithm embeds a binary variable interacting with the already embedded binary variables into the graph. The latter procedure is iterated until all qubits in the Chimera graph are used. It is generally necessary to assign several qubits to one binary variable and extend a chain to correctly represent interactions between binary variables on the Chimera graph. This algorithm implements Dijkstra’s algorithm to greedily determine how to extend chains. Although chains often cannot be extended by only unused qubits, this difficulty can be avoided in the embedding of subproblems because it is not necessary to embed all binary variables. In this case, the algorithm stops attempting to embed the binary variable and attempts to embed other binary variables that can be easily embedded. Thus, extracting and embedding a subproblem is simultaneously implemented in this algorithm, and the resulting subproblem comprises only binary variables that can be easily embedded. Therefore, the computational time is significantly lower than in Cai’s algorithm^50^. In addition, this algorithm can easily deal with hardware defects by implementing Dijkstra’s algorithm on a Chimera graph with defects.

It is the random partition to directly apply this algorithm to extract and embed a subproblem of integer optimization problems because this algorithm does not address whether an extracted subproblem contains feasible solutions for each *S*_*i*_ or not. To combine the multivalued partition with this algorithm, the order of the binary variables embedded into the Chimera graph must be appropriately specified, as described in the following subsection.

### Multivalued partition

The multivalued partition requires that the binary variable assigned to the tentatively selected component must be embedded into the Chimera graph. In addition, more than two binary variables should be embedded for each integer variable to distinguish the multivalued and binary partitions. On the other hand, the subproblem-embedding algorithm extracts and embeds a subproblem comprising binary variables that can be easily embedded. Therefore, in order to combine the multivalued partition and the subproblem-embedding algorithm, it is needed to appropriately specify the order of the binary variables embedded into the Chimera graph.

First, an integer variable is randomly selected, and binary variables assigned to the selected integer variable are embedded into the Chimera graph. Then, to determine binary variables to be additionally embedded into the Chimera graph, we select an integer variable as follows:

1. An already embedded binary variable *x*_embedded_ is selected in the order of being embedded into the Chimera graph.
2. An integer variable *S*_ctr_ to which *x*_embedded_ is assigned is selected.
3. Integer variables {*S*_*i*_} that interact with *S*_ctr_ in the problem graph are extracted.
4. An integer variable *S*_*i*_ is selected in a random order from {*S*_*i*_}.
5. The binary variables ${\{{x}_{i}^{(q)}\}}_{q=1,2,...,Q}$ assigned to *S*_*i*_ are attempted to be embedded.

Then, the order of the binary variables ${\{{x}_{i}^{(q)}\}}_{q=1,2,...,Q}$ embedded into the Chimera graph is determined using the following two criteria:

1. The binary variable adjacent to the binary variables that are already embedded.
2. The binary variable assigned to the tentatively selected component.

For the binary variable that is embedded first among ${\{{x}_{i}^{(q)}\}}_{q=1,2,...,Q}$, criterion 1 is important than criterion 2 to avoid embedding independent integer variables. For the reminder of the binary variables assigned to *S*_*i*_, we prioritize criterion 2 to achieve the multivalued partition. It should be noted that the number of components embedded into the Chimera graph is not uniform for each integer variable because the subproblem-embedding algorithm embeds only binary variables that can be easily embedded. If only one component can be embedded, the integer variable is excluded from the subproblem.

Table 3 represents the average number *N*_*S*_(*Q*_embed_) of embedded integer variables with *Q*_embed_ components per subproblem. The performance is assessed for embedding the Potts gauge glass model on the cubic lattice into D-Wave 2000Q_2 with defects and is averaged over 1,000 trials. 65.7(=14.5 + 8.0 + 43.2) integer variables are embedded into the Chimera graph on average, and the average number of embedded binary variables is as follows:

11 $$\mathop{\sum }\limits_{{Q}_{{\rm{embed}}}=1}^{4}{Q}_{{\rm{embed}}}{N}_{S}({Q}_{{\rm{embed}}})=2\times 14.5+3\times 8.0+4\times 43.2=225.8.$$

**Table 3:** Average number of embedded integer variables with *Q*_embed_ components.

*Q*_embed_	*N*_*S*_ (*Q*_embed_)
2	14.5
3	8.0
4	43.2

It should be noted that, to distinguish the multivalued and binary partitions, *Q*_embed_ > 2 is required for most integer variables. All four components are embedded for 65.8% of the integer variables in the subproblem, indicating that we can embed the multivalued subproblem that is distinct from the binary subproblem.

### Binary partition

To solve large optimization problems using the binary partition, the cost function of the binary subproblem must be derived from the cost function of the original large problem. The general form of the local energy between *S*_*i*_ and *S*_*j*_ is given by

12 $$\begin{array}{rcl}{H}_{ij} & = & \mathop{\sum }\limits_{q=1}^{Q}\mathop{\sum }\limits_{q^{\prime} =1}^{Q}{Q}_{ij}^{(qq^{\prime} )}{x}_{i}^{(q)}{x}_{j}^{(q^{\prime} )}+\mathop{\sum }\limits_{q=1}^{Q}({Q}_{ii}^{(qq)}{x}_{i}^{(q)}+{Q}_{jj}^{(qq)}{x}_{j}^{(q)})\\  &  & +\,\lambda {(\mathop{\sum }\limits_{q=1}^{Q}{x}_{i}^{(q)}-1)}^{2}+\lambda {(\mathop{\sum }\limits_{q=1}^{Q}{x}_{j}^{(q)}-1)}^{2},\end{array}$$

where ${Q}_{ij}^{(qq^{\prime} )}$ represents the interaction between ${x}_{i}^{(q)}$ and ${x}_{j}^{(q^{\prime} )}$. The binary partition extracts a binary subproblem by randomly selecting one component in addition to the tentatively selected component for each integer variable. The local energy of the binary subproblem in QUBO form is given as follows:

13 $${H}_{ij}^{({\rm{Binary}})}={R}_{ij}{y}_{i}{y}_{j}+{R}_{ii}{y}_{i}+{R}_{jj}{y}_{j},$$

14 $${R}_{ij}={Q}_{ij}^{({\alpha }_{i}{\alpha }_{j})}-{Q}_{ij}^{({\alpha }_{i}{\beta }_{j})}-{Q}_{ij}^{({\beta }_{i}{\alpha }_{j})}+{Q}_{ij}^{({\beta }_{i}{\beta }_{j})},$$

15 $${R}_{ii}=\sum _{k\ne i}({Q}_{ik}^{({\beta }_{i}{\alpha }_{k})}-{Q}_{ik}^{({\alpha }_{i}{\alpha }_{k})})-{Q}_{ii}^{({\alpha }_{i}{\alpha }_{i})}+{Q}_{ii}^{({\beta }_{i}{\beta }_{i})},$$

16 $${R}_{jj}=\sum _{k\ne j}({Q}_{kj}^{({\alpha }_{k}{\beta }_{j})}-{Q}_{kj}^{({\alpha }_{k}{\alpha }_{j})})-{Q}_{jj}^{({\alpha }_{j}{\alpha }_{j})}+{Q}_{jj}^{({\beta }_{j}{\beta }_{j})},$$

where *y*_*i*_ ∈ {0, 1}, *α*_*i*_ and *β*_*i*_ denote the tentatively selected component and randomly selected component for *S*_*i*_, respectively, and *y*_*i*_ = 0(*y*_*i*_ = 1) indicates “stay in the tentatively selected component *α*_*i*_” “(“transit to the other component *β*_*i*_”)” It should be noted that the cost function of the binary subproblem does not contain the penalty term because all solutions in the binary subproblem satisfy the one-hot constraint.

The problem graph of the binary subproblem extracted from the three-dimensional Potts model is a cubic lattice with bond dilutions. The density of the interactions in the binary subproblem is lower than that in the multivalued subproblem because the cost function of the binary subproblem does not contain the penalty term, which generates partially fully connected interactions between ${x}_{i}^{(q)}$ and ${x}_{i}^{(q^{\prime} )}$. The average number of embedded binary variables is 408 when the binary partition is used, and only 225 when the multivalued partition is used. Furthermore, all configurations in the binary subproblem satisfy the one-hot constraint, while the configurations in the multivalued subproblem do not. Therefore, the average number *N*_feasible_ of feasible solutions involved in the embedded subproblem is considerably increased using the binary partition. Table 4 illustrates log_10_*N*_feasible_ in a subproblem embedded by the multivalued and binary partitions combined with the complete graph embedding^44^ and subproblem-embedding algorithm^41^ for the Potts gauge glass model on the cubic lattice.

**Table 4:** Average number of feasible solutions in an embedded subproblem: log_10_*N*_feasible_.

	Complete graph embedding	Subproblem-embedding algorithm
Multivalued partition	9.6	33.9
Binary partition	19.3	122.8
